# Breastfeeding and Future Cardiovascular, Kidney, and Metabolic Health—A Narrative Review

**DOI:** 10.3390/nu17060995

**Published:** 2025-03-12

**Authors:** You-Lin Tain, Ying-Jui Lin, Chien-Ning Hsu

**Affiliations:** 1 Division of Pediatric Nephrology, Kaohsiung Chang Gung Memorial Hospital, Kaohsiung 833, Taiwan; tainyl@cgmh.org.tw; 2 College of Medicine, Chang Gung University, Taoyuan 333, Taiwan; 3 Department of Pediatrics, Kaohsiung Municipal Ta-Tung Hospital, Kaohsiung 801, Taiwan; 4 Division of Critical Care, Department of Pediatrics, Kaohsiung Chang Gung Memorial Hospital and Chang Gung University College of Medicine, Kaohsiung 833, Taiwan; rayray@cgmh.org.tw; 5 Division of Cardiology, Department of Pediatrics, Kaohsiung Chang Gung Memorial Hospital and Chang Gung University College of Medicine, Kaohsiung 833, Taiwan; 6 Department of Respiratory Therapy, Kaohsiung Chang Gung Memorial Hospital and Chang Gung University College of Medicine, Kaohsiung 833, Taiwan; 7 Department of Early Childhood Care and Education, Cheng Shiu University, Kaohsiung 833, Taiwan; 8 Department of Pharmacy, Kaohsiung Chang Gung Memorial Hospital, Kaohsiung 833, Taiwan; 9 School of Pharmacy, Kaohsiung Medical University, Kaohsiung 807, Taiwan

**Keywords:** breastfeeding, lactation, developmental origins of health and disease (DOHaD), microbiota, kidney disease, cardiovascular disease, metabolic syndrome, milk composition

## Abstract

The benefits of breastfeeding for both mother and infant are generally recognized; however, the connections between breast milk, lactation, and long-term offspring health and disease remain incompletely understood. Cardiovascular–kidney–metabolic syndrome (CKMS) has become a major global public health challenge. Insufficient breast milk supply, combined with various early-life environmental factors, markedly increases the future risk of CKMS, as highlighted by the developmental origins of health and disease (DOHaD) concept. Given its richness in nutrients and bioactive components essential for infant health, this review focuses on reprogramming strategies involving breast milk to improve offspring’s cardiovascular, kidney, and metabolic health. It also highlights recent experimental advances in understanding the mechanisms driving CKMS programming. Cumulatively, the evidence suggests that lactational impairment heightens the risk of CKMS development. In contrast, early interventions during the lactation period focused on animal models that leverage breast milk components in response to early-life cues show potential in improving cardiovascular, kidney, and metabolic outcomes—an area warranting further investigation and clinical translation.

## 1. Introduction

Lactation, a critical period during which the development of fragile organ systems continues, plays a key role in infant growth and occurs in all female mammals. It involves the secretion of milk from the mammary glands and the period during which a mother produces milk to nourish her offspring. In humans, this process is known as breastfeeding or nursing.

Breast milk is universally recognized as the ideal source of nutrition for infants [1,2,3]. It contains a rich array of nutrients and bioactive compounds, including hormones, growth factors, cytokines, microRNAs, metabolites, prebiotics, probiotics, and oligosaccharides [1]. These components have a key role in supporting and promoting offspring health. Conversely, compromised lactation during the early stages of life may increase the risk of insufficient development in infants and contribute to adverse outcomes later in life [4,5].

Adverse environmental conditions during early life can increase susceptibility to adult diseases, a concept known as the developmental origins of health and disease (DOHaD) [6,7]. This theory suggests that developmental programming responds to environmental challenges by undergoing structural and functional adaptations, which can elevate the risk of adulthood chronic diseases. While most studies on developmental programming focus on the prenatal stage, relatively few explore the long-term health consequences of challenges encountered during the breastfeeding period [8,9].

Cardiovascular–kidney–metabolic syndrome (CKMS) has become a critical global public health problem [10]. Though newly defined by the American Heart Association in 2023 [11], its prevalence is estimated to affect nearly 90% of the adult population in the United States [12]. The complex interconnections among cardiovascular disease (CVD), chronic kidney disease (CKD), and metabolic disorders have been extensively studied [13,14,15,16]. CKMS is a systemic disorder characterized by complex pathophysiological interactions among metabolic risk factors, kidney disease, and the cardiovascular system, leading to multi-organ dysfunction and an increased risk of adverse cardiovascular, metabolic, and renal outcomes [11]. CKMS is classified into four stages (0–4), reflecting varying degrees of disease progression and severity. Given the potential for multi-organ dysfunction resulting from early-life insults, early prevention—rather than focusing solely on treatment—offers promise for reducing the global burden of CKMS [17]. Emerging evidence suggests that interventions during critical developmental stages, such as lactation, may mitigate or even reverse adverse programming effects, a process termed reprogramming [18]. This underscores the potential of regulating maternal diet during the breastfeeding period as a reprogramming strategy to prevent CKMS.

Proper nutrition during breastfeeding is essential for the health of both mothers and their offspring [19]. While the beneficial effects of breastfeeding are well-established [1,2,3], our understanding of its role in the developmental programming of CKMS remains limited. This review explores the impact of breastfeeding on CKMS programming by synthesizing epidemiological and experimental evidence, focusing on breast milk components, and discusses potential interventions using breastfeeding as a reprogramming strategy to mitigate CKMS onset.

A comprehensive literature review was conducted by identifying relevant studies published in English through scientific databases, including MEDLINE, the Cochrane Library, and Embase. Our review includes clinical studies, observational studies, clinical trials, and animal research published between January 2000 and December 2024, with a focus on full-text articles in English. The search employed key terms related to breastfeeding, DOHaD, and CKMS. The employed search terms included “breastfeeding”, “lactation”, “metabolic syndrome”, “obesity”, “chronic kidney disease”, “hypertension”, “hyperlipidemia”, “dyslipidemia”, “insulin resistance”, “hyperglycemia”, “diabetes”, “liver steatosis”, “atherosclerosis”, “heart failure”, “cardiorenal syndrome”, “cardiovascular disease”, “developmental programming”, “DOHaD”, “offspring”, “progeny”, “mother”, “nitric oxide”, “oxidative stress”, “reprogramming”, “gut microbiota”, “milk composition”, and “breast milk”. Additionally, we carefully examined the reference lists of articles to identify any additional sources relevant to this review.

## 2. Lactation as a Developmental Window for CKMS Programming

Lactation is a critical postnatal period, and any alteration in breastfeeding or breast milk composition can adversely affect offspring health. Table 1 summarizes various environmental insults during lactation and/or gestation that are associated with CKMS [20,21,22,23,24,25,26,27,28,29,30,31,32,33,34,35,36,37,38,39,40,41,42,43,44,45,46,47,48,49,50,51,52,53,54,55,56,57,58,59,60,61,62,63,64,65,66,67]. Table 1 primarily includes observations from animal research demonstrating at least two components of CKMS in adult offspring. Notably, postnatal insults that directly affect offspring were excluded from this review, as they are likely independent of breastfeeding-related aspects.

Since CKMS was defined in 2023, no clinical evidence is yet available on the long-term impact of breastfeeding. All studies summarized in Table 1 are based on rodent models. After birth, one day in a rat’s life roughly corresponds to nine days in a human [68]. A three-week lactation period in rats is approximately equivalent to six months of breastfeeding in humans [68]. Additionally, one month of adulthood in rats is roughly equivalent to three human years. Table 1 also highlights that the outcomes are assessed from 12 to 52 weeks of rat age, aligning with the developmental period from adolescence to middle adulthood in humans. Various early-life factors have been implicated in inducing CKMS in adult offspring, including maternal malnutrition, medical conditions, pregnancy complications, environmental chemical exposures, and drug use.

### 2.1. Maternal Malnutrition

Maternal nutrition determines the nutritional composition of breast milk [69]. Table 1 highlights that maternal nutritional imbalances during lactation and/or gestation play a crucial role in CKMS programming. These imbalances include low-calorie diets [20,21,22], low-protein diets [23,24], high-fructose diets [25,26,27,28], high-fat diets [29,30,31,32], and over-nutrition [33,34,35]. Notably, litter size reduction during the lactation period was initially used as an experimental method to induce overfeeding, accelerated neonatal growth, and early onset of overweight/obesity in rodents [70]. Unlike other maternal diet-induced nutritional imbalance models, the programming effects of litter size reduction are confined to the breastfeeding period. As shown in Table 1, most CKMS components, such as hypertension, obesity, insulin resistance, and kidney disease [33,34,35], can be induced using this model, underscoring the critical role of breastfeeding in CKMS programming.

### 2.2. Maternal Medical Condition and Pregnancy Complication

Additionally, maternal medical conditions and pregnancy complications can interfere with breastfeeding, contributing to the development of CKMS later in life. For example, diabetes can affect breastfeeding by delaying the establishment of milk production, altering maternal blood sugar patterns, and shortening the breastfeeding duration [71]. Maternal diabetes has been associated with the onset of hypertension, obesity, insulin resistance, dyslipidemia, and kidney disease in adult offspring [36,37,38], all of which are key components of CKMS. Uteroplacental insufficiency, a common pregnancy complication, can impair mammary gland development in mother rats, leading to reduced milk production with altered composition and contributing to various features of CKMS [39,40,41,42,43,44].

Several maternal conditions have also been associated with the development of CKMS in offspring, including maternal chronic kidney disease (CKD) [44,45], maternal stress [46,47,48,49], maternal hypoprolactinemia [50,51], nicotine exposure [52,53,54,55], and ethanol exposure [56,57]. All these conditions are closely related to breastfeeding [1,72,73,74].

### 2.3. Environmental Pollutants Exposure

Furthermore, breast milk can contain environmental pollutants. Environmental pollutants can act as endocrine-disrupting chemicals (EDCs), affecting breastfeeding by disrupting mammary gland development [75]. Numerous epidemiological studies have examined the relationships between background levels of these pollutants in breast milk and health outcomes in infants and children [76]. As shown in Table 1, maternal exposure to bisphenol A (BPA) [58,59,60,61], 2,3,7,8-tetrachlorodibenzo-*p*-dioxin (TCDD) [62,63,64], and di-*n*-butyl phthalate (DEHP) [65,66,67] has been linked to the development of CKMS in adult rodent progeny.

### 2.4. Drug Use

Maternal use of cyclosporine A [77], gentamicin [78], and glucocorticoids [79] has been associated with a reduced nephron number, which is linked to kidney disease and hypertension in adulthood. However, their programming effects on cardiovascular and metabolic outcomes remain unclear and have not been thoroughly evaluated. Additionally, a study indicated that the use of metformin during the lactation period induces metabolic programming [80], but its effects on the kidney and cardiovascular systems are yet to be explored.

Drugs taken by a breastfeeding mother can transfer into breast milk in varying amounts, potentially affecting the infant. However, most drugs, such as non-steroidal anti-inflammatory drugs (NSAIDs)—commonly used by lactating women—are generally considered safe due to their low milk transfer [81]. Similarly, some antiepileptic drugs are regarded as safe for use during lactation [82]. Nevertheless, the long-term effects of these medications on offspring are insufficiently studied and remain inadequately reported in both clinical and experimental research.

## 3. Breast Milk: Synthesis and Components

Breast milk synthesis is a complex process that depends on the proper development and differentiation of the mammary gland, as well as the availability of essential nutrients and elements [83]. Prolactin has a vital role in stimulating the synthesis of milk proteins and lactose [84]. Endogenously produced substances such as major milk proteins, oligosaccharides, lactose, calcium, and phosphate are secreted via the exocytic pathway, with secretory vesicles transporting them from the Golgi apparatus to the cell membrane for release. Lipids and lipid-like proteins, synthesized in mammary alveolar cells, are secreted as membrane-enveloped milk fat globules through a specialized budding process. Transport pathways enable the transfer of amino acids, ions, glucose, and trace elements from the bloodstream to milk through coordinated apical and basal plasma membranes and transport proteins.

Breast milk offers the optimal balance of nutrients and bioactive compounds needed for the developing infant, along with beneficial microbes that help protect the immature immune system against disease [85]. Each of these components in human breast milk will be discussed in detail.

### 3.1. Macronutrients

Breast milk evolves throughout lactation, from colostrum to transitional to mature milk [1]. Colostrum, produced in small quantities during the first 2–4 days postpartum, is distinct in color, composition, and consistency. It is rich in whey proteins, sodium, chloride, minerals, and magnesium but has lower levels of fats, lactose, potassium, calcium, and certain vitamins than mature milk [86]. Transitional milk, produced from 5 days to 2 weeks postpartum, supports the infant’s growth, while mature milk, produced after 4 weeks after postpartum, stabilizes in composition but undergoes slight nutrient fluctuations.

Mature milk typically contains approximately 3–5% fat, 6.9–7.2% lactose, 0.8–0.9% protein, and 0.2% minerals. The energy content of human breast milk is approximately 67 kcal per 100 mL, with fat (~3.2–4.3 g per 100 mL) providing ~50% of total energy, carbohydrates (mainly lactose, ~6.7–7.8 g per 100 mL) contributing ~40%, and protein (~0.9–1.2 g per 100 mL) accounting for ~10% of energy. Key proteins include casein, IgA, lactoferrin, α-lactalbumin, serum albumin, and lysozyme [87]. Fat, the primary energy source, varies in concentrations during feeding; hindmilk is richer in fat than foremilk. This composition is also influenced by maternal diet and parity [88,89]. One study found that women with overweight or obesity had significantly higher fat and calorie content in both their first foremilk and last hindmilk compared to women with normal BMI, potentially influencing infant growth and the risk of childhood obesity [90].

Unlike protein and fat, lactose levels remain relatively stable over time, including in colostrum [91]. Human research indicates that maternal consumption of sugar-sweetened beverages (SSBs) containing fructose leads to higher fructose levels in breast milk. This increase is associated with greater body weight, fat mass, and lean mass in infants at six months. These findings underscore the need for dietary recommendations for breastfeeding mothers, as fructose from SSBs is not a natural component of breast milk [92,93].

In addition, human milk oligosaccharides (HMOs) play a critical role in shaping the development of an infant’s gut microbiota [94]. More than 200 HMOs have been identified to date, and their structure and composition in milk vary throughout the course of lactation [95]. These complex carbohydrates, the third most abundant solid component in human breast milk after lactose and lipids, act as prebiotics by selectively promoting the growth of beneficial gut bacteria such as *Bifidobacteria* and *Lactobacilli* [96,97]. HMOs also serve as decoy receptors to prevent pathogen attachment, support immune system development, and contribute to overall infant health and development.

### 3.2. Micronutrients

Human breast milk generally provides sufficient micronutrients for infant growth, though vitamins D and K may be inadequate [98,99]. Vitamin D levels in breast milk are low and depend on maternal sun exposure, diet, and lifestyle, with recommendations for lactating mothers and infants to take 200–400 IU/day of vitamin D, or 2000 IU/day in cases of deficiency [98]. Newborns have low vitamin K stores at birth, and unlike formula, breast milk contains low amounts of vitamin K [99]. Therefore, vitamin K supplementation is recommended after birth [99]. Water-soluble vitamins such as B6, B12, and folate may be deficient in mothers with low socioeconomic status [100]. In contrast, most minerals, including iron, copper, and zinc, are less influenced by maternal conditions and exhibit minimal variation with maternal supplementation [101]. Iron supplementation is generally unnecessary until 6 months of age, after which iron-containing drops are recommended [102].

### 3.3. MicroRNAs

MicroRNAs (miRNAs), a newly discovered class of bioactive components in human breast milk, are small, non-coding RNAs that regulate gene expression by inhibiting mRNA translation [103,104]. Among various body fluids, human milk is one of the richest sources of miRNAs [105]. These miRNAs, which influence key physiological functions, are encapsulated in extracellular vesicles, primarily exosomes, protecting them from digestive degradation and enabling absorption by intestinal cells [106]. Maternal factors such as nutrition, exercise, and diseases like obesity and diabetes can influence the abundance and composition of milk miRNAs [106]. Studies suggest that maternal obesity alters the miRNA profile in breast milk, potentially impacting infant growth and body composition [107,108]. Emerging evidence highlights the role of epigenetic regulation in breast milk, which has a crucial part in shaping infant health and susceptibility to diseases, including components of CKMS [109,110,111].

The most abundant miRNAs in human milk are miRNA-148a, followed by miRNA-21, miRNA-26a, miRNA-30a, miRNA-146b, miRNA-200a, miRNA-200c, and miRNA-146a [112,113]. Among these, miRNA-148a has been extensively studied and shown to play a significant role in regulating infant energy balance, adipogenesis, and lipid metabolism during breastfeeding [114].

### 3.4. Hormones and Growth Factors

Human breast milk contains various bioactive hormones and growth factors [1]. Hormones such as parathyroid hormone, melatonin, insulin, leptin, ghrelin, and adiponectin contribute to infant development, while growth factors like epidermal growth factor, vascular endothelial growth factor (VEGF), and insulin-like growth factors (IGF-1 and IGF-2) impact the cardiovascular system, kidneys, and metabolic processes [115,116]. For instance, melatonin, transferred from the mother’s plasma to breast milk, exposes the infant to circadian cues from the mother, aiding in the regulation of sleep–wake cycles [117]. Erythropoietin (EPO), primarily produced in the kidneys, promotes red blood cell production. A systematic review indicates moderate- to low-quality evidence suggesting that early prophylactic EPO reduces the risk of necrotizing enterocolitis in preterm neonates [118]. Furthermore, IGF-1 and VEGF play pivotal roles in angiogenesis and the prevention of retinopathy of prematurity, a condition that can affect preterm infants [119]. High levels of adiponectin regulate metabolism and are negatively associated with growth in infancy [120]. These bioactive components play critical roles in supporting infant health and development.

### 3.5. Breast Milk Microbiota

Human breast milk contains a diverse array of bacterial species, forming its own unique microbiome [85]. This microbiome plays a critical role in inoculating the infant’s gut with beneficial microbes after birth. Since an infant’s microbiota is ecologically shaped by maternal influence and breast milk, targeted management and nutritional interventions in the breast milk microbiome have the potential to enhance health not only during infancy but throughout life [121,122].

While the exact composition of the breast milk microbiota varies between mothers and across studies, a systematic review identified a dominance of *Staphylococcus*, *Streptococcus*, and *Lactococcus*, with a notable presence of *Pseudomonas*, *Lactobacilli*, and *Bifidobacteria* in colostrum and transitional milk [123]. Several factors likely influence the composition of the breast milk microbiota, including maternal diet, BMI, pregnancy weight gain, mode of delivery, and the use of prebiotics [123,124,125].

### 3.6. Others

Lactoferrin, the second most abundant protein in breast milk, is an iron-binding glycoprotein with antimicrobial properties that helps prevent neonatal sepsis, diarrhea, and necrotizing enterocolitis, particularly in pre-term infants. Its levels peak in colostrum (7 g/L) and decrease to 2–4 g/L in mature milk [126,127]. Human breast milk also contains secretory IgA and IgG, which provide immune protection [128].

Metabolomic studies have identified numerous metabolites in breast milk, including carbohydrates, amino acids, fatty acids, and nucleotides [129]. Previous work revealed that gestational diabetes is associated with altered levels of specific metabolites, such as elevated 2-hydroxybutyric acid and 3-methylphenylacetic acid and reduced 4-cresyl sulfate and glycine, among others [130]. Additionally, maternal obesity has been linked to 111 metabolites in breast milk, some of which, like mannose, lyxitol, and shikimic acid, are associated with infant adiposity, suggesting that these metabolites may mediate mother-to-child transmission of obesity risk [131].

While breast milk research continues to advance, understanding how maternal conditions and phenotypes affect milk composition and their subsequent impact on infant cardiovascular, kidney, and metabolic outcomes remains unclear. Despite progress in infant formula development, it cannot replicate the dynamic, evolving nature of human milk, which is uniquely tailored to meet an infant’s needs [132].

## 4. The Link Between Breastfeeding and Mechanisms Underlying CKMS Programming

Various early-life cues can lead to different manifestations of CKMS in adult offspring, suggesting that common mechanistic pathways may contribute to the pathogenesis of CKMS programming. Reported mechanisms include oxidative stress, dysregulation of the renin–angiotensin system (RAS), epigenetic modifications, inflammation, gut microbiota dysbiosis, and sex differences [17,18,133,134,135,136,137,138]. Some of these mechanisms are linked to breastfeeding and will be discussed further.

### 4.1. Oxidative Stress

Increased oxidative stress and reduced antioxidant defenses may be involved in the pathogenesis of perinatal disorders, with breastfeeding providing potential antioxidative protection [139]. A wide range of oxidative stress mechanisms have been recognized in CKMS programming, including the upregulated expression of reactive oxygen species (ROS)-generating enzymes, elevated ROS production, decreased antioxidant availability, enhanced oxidative damage, and disruption of the nitric oxide (NO) pathway.

Elevated abundance of ROS-producing enzymes and heightened ROS production have been observed in various CKMS programming models, such as those induced by maternal high-fructose diets [32], maternal stress [49], and nicotine exposure [52]. In the litter size reduction model, rat offspring exhibited obesity and insulin resistance, accompanied by reduced antioxidant enzyme activity, including catalase, superoxide dismutase, and glutathione peroxidase [34].

Several biomarkers indicative of oxidative damage to proteins, lipids, and DNA have also been assessed in animal models of CKMS programming. These include malondialdehyde (MDA) [32,53], 8-hydroxydeoxyguanosine (8-OHdG) [44], and 4-hydroxynonenal (4-HNE) [53]. Another key contributor to oxidative stress in CKMS pathogenesis is the impaired nitric oxide synthase (NOS)/NO pathway [140]. High levels of the endogenous NOS inhibitor, asymmetric dimethylarginine (ADMA), reduce NO production, leading to endothelial dysfunction and exacerbating oxidative stress. This has been linked to the development of various CKMS components in models of a low-calorie diet [22], maternal diabetes [36], maternal CKD [44], maternal stress [46], and perinatal BPA exposure [58].

### 4.2. Epigenetic Modifications

Epigenetic modifications, including miRNAs, histone modifications, and DNA methylation, play a pivotal role in regulating gene expression, a key mechanism underlying developmental programming [141]. Emerging evidence underscores the systemic bioavailability of exosome-derived miRNAs in breast milk and their significant gene-regulatory functions [142]. The developmental period from gestation to breastfeeding is characterized by increased epigenetic DNA imprinting activity [142,143]. Moreover, the breastfeeding period in infants serves as a crucial window for epigenetic development [143].

Abnormal DNA methylation patterns significantly impact breast milk synthesis [144]. Moreover, DNA methylation has been shown to correlate with gut microbiota profiles, which are closely linked to lipid metabolism and obesity [145]. In a mouse model with reduced dietary cholesterol availability from maternal milk, histone H3K9me3 methylation was increased, resulting in decreased expression of the intestinal cholesterol absorption transporter NPC1L1. This finding highlights the role of histone modifications in metabolic programming [146].

### 4.3. Gut Microbiota Dysbiosis

Emerging human evidence underscores the critical role of the gut microbiome in various components of CKMS, including CVD, CKD, obesity, and metabolic syndrome [147,148,149,150,151]. Nutrients in breast milk interact with gut microbes to produce critical metabolites, including short-chain fatty acids (SCFAs), trimethylamine-*N*-oxide (TMAO), and microbiota-derived uremic toxins, all of which are associated with CKMS [152,153,154].

Although microbial colonization of the human neonatal gut begins immediately after birth [155], the gut microbiome continues to develop and diversify in species abundance throughout infancy, achieving an adult-like composition by approximately two years of age, encompassing the lactation period [156]. The establishment of the gut microbiome is influenced by various maternal factors, including gestational age, mode of delivery, maternal health conditions, antibiotic exposure, and environmental factors [157]. These maternal influences are closely tied to breastfeeding practices and the composition of breast milk [1]. As a result, breastfeeding may act as a crucial link between maternal risk factors and the developmental programming of adult health and disease [158].

Conversely, interventions targeting the gut microbiota, such as probiotics, prebiotics, and postbiotics, have demonstrated health benefits in managing CKMS traits [159,160,161]. Notably, human breast milk contains a diverse array of bacterial species and HMOs that play an essential role in inoculating the infant’s gut with beneficial bacteria and prebiotics after birth [85,119,120]. Specifically, HMOs act as selective substrates for beneficial bacteria like *Bifidobacterium*, fostering a healthy gut environment that may have long-term benefits for offspring health. Given that an infant’s microbiota is ecologically shaped by the breast milk microbiota and its components, particularly HMOs, advancing our understanding of the mechanisms driving breast milk–gut microbiota interactions could provide valuable insights for developing microbiota-targeted interventions to mitigate the risk of CKMS.

### 4.4. Others

Given the multifaceted biological roles of breast milk, additional mechanisms, including its influence on inflammation, may contribute to its protective effects [162]. The accumulation of T cells and pro-inflammatory cytokines is a critical factor in the pathogenesis of various features of CKMS [163,164,165]. While growing evidence supports the involvement of inflammation in CKMS, limited research has specifically explored the anti-inflammatory actions of breast milk in preventing CKMS by targeting inflammation.

Additionally, sex differences are increasingly recognized as important mechanisms in fetal programming [166,167,168], and offspring sex has been shown to influence the nutrient and energy content of maternal milk [169]. However, the connections between these factors and CKMS programming remain unexplored, highlighting the need for further research in this area.

## 5. Targeting Breast Milk as a Reprogramming Strategy

Early-life reprogramming strategies to mitigate the mechanisms associated with DOHaD include avoiding risk factors, implementing nutritional interventions, utilizing pharmacological therapies, and promoting lifestyle modifications [17,18,132,170,171]. With significant advancements in understanding the benefits of breast milk and the mechanisms underlying CKMS programming in recent years, there is a pressing need to develop innovative reprogramming strategies that leverage breast milk as a preventive measure against CKMS.

Breast milk is widely recognized for its valuable nutrients and bioactive compounds, which are essential for the developing infant. These components have been linked to improved survival rates and notable cardiovascular, kidney, and metabolic benefits, particularly in high-risk infants [172,173]. Despite this, there is limited information from human and animal studies regarding the long-term reprogramming effects of breast milk, specifically concerning CKMS outcomes in later life [174].

Breastfeeding has been linked to a reduced risk of chronic conditions such as obesity, hypertension, dyslipidemia, and diabetes mellitus in adulthood. However, the existing literature remains inconclusive, and the underlying protective mechanisms are not yet fully elucidated [175,176,177,178]. Table 2 summarizes findings from rodent studies investigating the effects of breast milk components administered during lactation and/or gestation on offspring CKMS phenotypes, with a focus on interventions implemented before the onset of clinical symptoms [36,179,180,181,182,183,184,185,186,187,188,189,190,191,192,193,194,195,196,197].

Various CKMS programming models have been studied, utilizing diverse approaches such as low-protein diets [179,184], low-calorie diets [189,196], maternal diabetes [36,193], maternal CKD [179,191], maternal dyslipidemia [181], maternal high-fat diets [182,183,188,195], cafeteria diets [185], high-salt diets [186], maternal LPS exposure [187], Western diets [190], high-fat/sucrose diets [192], tryptophan-free diets [194], and neonatal corticosterone exposure [197]. These studies primarily focus on evaluating components of CKMS, with hypertension being the most studied phenotype, followed by obesity, kidney disease, diabetes, fatty liver, cardiovascular disease, and dyslipidemia. Protective effects against CKMS have been detected in rats aged 8 to 56 weeks, approximately corresponding to human ages from adolescence to middle adulthood.

Nutritional and bioactive components of breast milk employed as reprogramming interventions include amino acids, fats, micronutrients, hormones, HMOs, SCFAs, and phytochemicals. The following sections will explore each of these components in detail.

### 5.1. Amino Acids

Amino acids are amongst the most variable metabolites in human milk across all lactation phases and can vary significantly between mothers [198,199]. Considering the importance of amino acids implicated in developmental programming, the supplementation of amino acids during pregnancy has been examined to prevent CKMS components in animal models, as reviewed elsewhere [200]. As indicated in Table 2, supplementation with L-arginine, L-citrulline, L-taurine, and L-leucine during the lactation period has been used to protect offspring against hypertension, kidney disease, obesity, dyslipidemia, and glucose intolerance [36,179,180,181,182].

Previous studies demonstrated that supplementation with L-arginine during the lactation period protected adult offspring from metabolic programming in a low-protein diet rat model [179]. L-citrulline, a precursor that increases plasma L-arginine levels and enhances NO production, has gained attention for its ability to bypass hepatic metabolism and convert to L-arginine in the kidneys [180]. L-citrulline supplementation during lactation and gestation has been shown to protect offspring from hypertension induced by maternal diabetes [38].

Similarly, perinatal L-taurine treatment in the context of maternal CKD effectively protects offspring from hypertension [180]. In a maternal hyperlipidemia rat model, perinatal L-taurine supplementation improved outcomes related to obesity, dyslipidemia, and hypertension in offspring [181]. Additionally, perinatal L-leucine supplementation was shown to mitigate obesity and glucose intolerance in adult mouse offspring exposed to a maternal high-fat diet [182].

### 5.2. Fats

Breast milk fats and their downstream fatty acid derivatives not only serve as an energy source but also play key roles in regulating essential physiological functions [201]. While saturated and *trans* fats increase CVD risk, monounsaturated and polyunsaturated fatty acids (PUFAs), including linoleic and alpha-linolenic acids found in human milk, are linked to a decreased CVD risk [202,203]. As detailed in Table 2, supplementation with conjugated linoleic acid (CLA) and PUFAs has been employed as reprogramming interventions to address offspring CKMS [183,184,185]. CLA contains one or more *trans* double bonds in its fatty acid structure, classifying it as a naturally occurring *trans* fat. However, unlike harmful industrial *trans* fats (e.g., partially hydrogenated oils) associated with CVD, CLA is naturally present in meat and dairy products and has been studied for potential health benefits. CLA, derived from dietary PUFAs like linoleic acid, has been shown to protect adult rat offspring from hypertension induced by a high-fat diet when supplemented during breastfeeding [183]. Additionally, perinatal PUFA supplementation has demonstrated protective effects against hypertension and CVD in adult rat progeny born to dams subjected to protein restriction [184], as well as against fatty liver in offspring from a maternal cafeteria diet model [185].

Despite recommendations for pregnant and breastfeeding women to consume PUFAs [204], a meta-analysis of 3644 children found no significant reduction in obesity risk from maternal omega-3 PUFA supplementation during pregnancy [205]. As a result, the impact of specific fats during breastfeeding on offspring CKMS risk remains inconclusive.

### 5.3. Micronutrients

The antioxidant capacity of breast milk includes vitamins (A, C, and E) and antioxidant enzymes [1,139]. Vitamin E, a lipid-soluble antioxidant, inhibits various oxidative enzymes, thereby reducing ROS production [206]. As noted in Table 2, supplementation with vitamin E during the lactation period has been revealed to protect against offspring hypertension and CVD induced by a maternal high-salt diet [186]. Folic acid, an essential water-soluble B vitamin, plays a critical role during breastfeeding due to its involvement in one-carbon metabolism [207]. One-carbon metabolites serve as methyl donors necessary for DNA methylation. In CD-1 mice exposed to lipopolysaccharide (LPS), folic acid supplementation during lactation and gestation confirmed protective effects against the development of glucose intolerance in adult offspring [187]. Similarly, perinatal betaine supplementation was shown to attenuate fatty liver in adult mice offspring born to dams fed high-fat diets [188]. Betaine is commonly used as an alternative methyl donor, further underscoring the role of one-carbon metabolism in CKMS programming.

### 5.4. Bioactive Components

Several bioactive components have been explored in the context of CKMS programming. Previous studies have shown that growth hormone therapy for mothers experiencing lactational insufficiency can enhance breast milk production [208]. Furthermore, supplementation with growth hormone during the lactation period has been found to improve offspring outcomes, including mitigating hypertension and cardiovascular disease induced by a maternal low-calorie diet [189].

Leptin, an adipocyte-derived hormone, plays a pivotal role during breastfeeding by regulating energy homeostasis and influencing susceptibility to obesity later in life [209]. In a maternal Western diet model, leptin supplementation during breastfeeding protected adult offspring from obesity and insulin resistance [190]. Lactoferrin, a multifunctional glycoprotein predominantly found in colostrum, is another critical component of breast milk [210]. As summarized in Table 2, maternal lactoferrin treatment has been shown to counteract maternal CKD-induced offspring hypertension by enhancing nitric oxide (NO) availability and modulating gut microbiota composition [191].

HMOs have been receiving significant attention for their role in shaping the microbiota during early development [94]. Perinatal supplementation with oligofructose has been reported to protect adult rat offspring from diabetes and fatty liver associated with a maternal high-fat/sucrose diet [192]. The SCFAs in breast milk also contribute to infant health by enhancing intestinal immunity and promoting a healthy microbiota [211]. Among SCFAs, butyrate is particularly noteworthy for its cardiovascular and metabolic benefits [212]. Perinatal butyrate supplementation has demonstrated protective effects against diabetes in an NOD mouse model and hypertension in a tryptophan-deficient diet model [193,194].

Additionally, phytochemicals, including polyphenols and carotenoids, are valued for their potent antioxidant properties and presence in breast milk [213]. While maternal intake of polyphenols has been linked to benefits for offspring’s cardiovascular, kidney, and metabolic health [214], only a subset of these compounds has been detected in breast milk [215,216]. In a maternal high-fat diet model, perinatal quercetin supplementation improved multiple components of CKMS in adult rat offspring, including obesity, hyperglycemia, hyperlipidemia, and hyperinsulinemia [195].

Melatonin in breast milk plays a crucial role in regulating an infant’s circadian cycle. Its concentration follows a consistent pattern, being higher at night and particularly elevated in colostrum [217]. This hormone also plays a multifaceted role in developmental programming [218,219], making it a promising candidate for reprogramming strategies targeting CKMS [220]. In a neonatal corticosterone exposure model, melatonin treatment during the lactation period successfully prevented adverse metabolic programming [196]. Corticosterone-programmed rats, which typically develop hyperglycemia, hyperlipidemia, liver steatosis, and diabetes, were protected from these outcomes through melatonin therapy [196]. Additionally, perinatal melatonin treatment prevented hypertension and kidney disease in offspring resulting from a maternal low-calorie diet [197].

**Figure 1:**
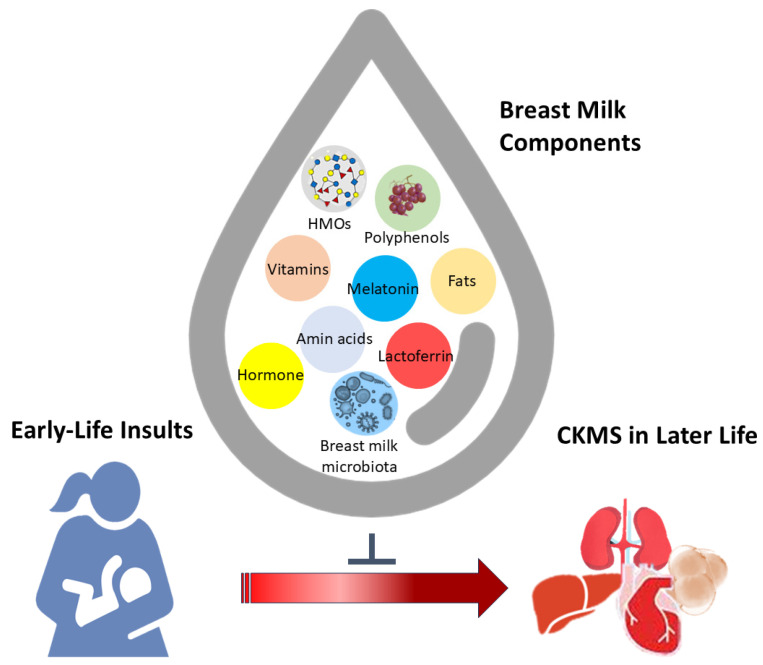
A visual representation of how breastfeeding and breast milk components contribute to preventing the developmental programming of cardiovascular–kidney–metabolic syndrome (CKMS) in offspring later in life.

While breast milk contains numerous bioactive components, only a few have been studied for their reprogramming effects on CKMS. For instance, the epigenetic role of breast milk through miRNAs remains largely unclear. Oral supplementation with miRNA-320-3p or miRNA-375-3p during the lactation period has demonstrated long-term, miRNA-specific effects on endogenous miRNA levels, exhibiting strong tissue-dependent memory [221]. However, additional studies are needed to investigate their long-term impacts on offspring’s cardiovascular, kidney, and metabolic health outcomes. Figure 1 highlights the complex interplay among breastfeeding, the nutrients and bioactive components in breast milk, developmental programming, and offspring CKMS, as outlined in this review.

## 6. Concluding Remarks and Perspectives

Can breastfeeding influence lifelong health? While the benefits of breastfeeding are now well recognized, its role in CKMS programming remains underexplored. Despite CKMS and its associated disorders emerging as major public health concerns, specific preventive interventions are still lacking [222]. Lactational impairment, which encompasses difficulties or abnormalities in the process of lactation that affect the production, composition, or secretion of breast milk in clinical practice, along with various early-life environmental factors, significantly influences the future risk of CKMS. Conversely, animal studies suggest that certain nutrients and bioactive components in breast milk may serve as effective reprogramming agents to support cardiovascular, kidney, and metabolic health. However, a key limitation of this review is the inability to isolate lactation-specific effects, as most studies encompass both gestation and lactation. Additionally, while similar factors influence human milk, no direct data link it to CKMS development beyond hypothetical associations. Moreover, these findings are primarily from animal studies, and a direct correlation to human infants is not possible. Many questions remain regarding their translation into clinical practice.

First, gathering definitive data on the role of specific nutrients and bioactive components in breast milk, as well as their interactions in CKMS programming, is a major challenge in this field. Second, long-term clinical outcomes related to CKMS screening, staging, and therapy remain unclear, and since CKMS was only defined in 2023, there is an urgent need for this information to develop effective early prevention strategies. Third, while numerous animal studies highlight the benefits of reprogramming interventions on cardiovascular, kidney, and metabolic health, only a few focus exclusively on the lactation period. These effects likely extend across various developmental windows, leaving their true impact uncertain. Lastly, despite advancements in identifying bioactive components in breast milk, there has been limited exploration of their specific roles in reprogramming each element of CKMS. Additionally, this review does not address the impact of formula feeding on CKMS programming. Although current evidence suggests that many bioactive components can be added to formula milk to confer potential benefits [132], further research is needed.

Understanding the distinct mechanisms of breastfeeding and identifying the specific breast milk components that influence CKMS developmental programming are crucial. There is optimism that interventions during the lactation period could promote healthier developmental trajectories and improve future cardiovascular, kidney, and metabolic health in the future.

## Figures and Tables

**Table 1:** Summary of environmental insults during lactation and/or gestation linked to cardiovascular–kidney–metabolic syndrome in rodent models.

Model	Exposure Timing Gestation/Lactation	Age at Evaluation (Weeks)	Components of CKMS	References
Low caloric diet	Yes/Yes	12–16	Insulin resistance, hypertension, and kidney disease	[20,21,22]
Low protein diet	Yes/Yes	16–24	Insulin resistance, hypertension, and kidney disease	[23,24]
High-fat diet	Yes/Yes	16	Obesity, insulin resistance, dyslipidemia, hypertension, and kidney disease	[25,26,27,28]
High-fructose diet	Yes/Yes	12–52	Hypertension, obesity, insulin resistance, and dyslipidemia	[29,30,31,32]
Litter size reduction	No/Yes	24–52	Obesity, insulin resistance, hypertension, and kidney disease	[33,34,35]
Maternal diabetes	Yes/Yes	12–16	Hypertension, obesity, insulin resistance, dyslipidemia, and kidney disease	[36,37,38]
Uteroplacental insufficiency	Yes/No	22–30	Dyslipidemia, insulin resistance, hypertension, and kidney disease	[39,40,41,42,43,44]
Maternal chronic kidney disease	Yes/Yes	12	Kidney disease and hypertension	[44,45]
Maternal stress	Yes/Yes	16–24	Obesity, insulin resistance, hypertension, and kidney disease	[46,47,48,49]
Maternal hypoprolactinemia	No/Yes	13–24	Obesity, insulin resistance, and kidney disease	[50,51]
Nicotine exposure	Yes/Yes	20–32	Hypertension, kidney disease, steatosis, and hyperlipidemia,	[52,53,54,55]
Ethanol exposure	Yes/Yes	12–24	Insulin resistance and cardiovascular disease	[56,57]
BPA exposure	Yes/Yes	16–24	Hypertension, insulin resistance, steatosis, and cardiovascular disease	[58,59,60,61]
TCDD exposure	Yes/Yes	12	Hypertension, kidney disease, and cardiovascular disease	[62,63,64]
DEHP exposure	Yes/Yes	12–21	Hypertension and insulin resistance	[65,66,67]

BPA = bisphenol A; TCDD = 2,3,7,8-tetrachlorodibenzo-*p*-dioxin; DEHP = di-*n*-butyl phthalate.

**Table 2:** Nutritional and bioactive component supplementation from breast milk for the prevention of cardiovascular–kidney–metabolic syndrome phenotypes in rodent models.

Reprogramming Intervention	Period Gestation/Lactation	Experimental Model	Species	Age at Evaluation (Weeks)	Protective CKMS Phenotype	Ref
Amino acids						
L-arginine (200 mg/kg/day)	No/Yes	Low protein diet	SD rat/M	8	Hepatic insulin signaling	[179]
L-citrulline (2.5 g/L in drinking water)	Yes/Yes	Maternal diabetes	SD rat/M	12	Hypertension and kidney disease	[36]
L-taurine (3% in drinking water)	Yes/Yes	Maternal CKD	SD rat/M	12	Hypertension and kidney disease	[180]
L-taurine (3% in drinking water)	Yes/Yes	Maternal dyslipidemia	Wistar rat/M and F	16	Obesity, dyslipidemia, and hypertension	[181]
L-leucine (1.5% in chow)	Yes/Yes	High-fat diet	C57BL/6 mice/M	16	Obesity and glucose intolerance	[182]
Fats						
Conjugated linoleic acid (1% in chow)	Yes/Yes	Hight-fat diet	SD rat/M	18	Hypertension	[183]
PUFA (1.5 g/kg/day)	Yes/Yes	Low protein diet	Wistar rat/M and F	24	Hypertension and cardiovascular disease	[184]
PUFA (8.78% in chow)	Yes/Yes	Cafeteria diet	SD rat/M	56	Fatty liver	[185]
Micronutrients						
Vitamin E (0.35 g/kg/day)	No/Yes	High-salt diet	Wistar rat/M	30	Kidney disease and cardiovascular disease	[186]
Folic acid (5 mg/kg/day)	Yes/Yes	Maternal LPS exposure	CD-1 mice/M	16	Glucose intolerance	[187]
Betaine (1% in drinking water)	Yes/Yes	High-fat diet	C57BL/6 mice/M and F	8	Fatty liver	[188]
Bioactive components						
Growth hormone (2.5 ug/g/day)	No/Yes	Low caloric diet	SD rat/M	23	Hypertension and cardiovascular disease	[189]
Leptin ^1^	No/Yes	Western diet	Wistar rat/M	16	Obesity and insulin resistance	[190]
Lactoferrin (10% in chow)	Yes/Yes	Maternal CKD	SD rat/M	12	Hypertension	[191]
Oligofructose (10% in drinking water)	Yes/Yes	High-fat/sucrose diet	SD rat/M	24	Diabetes and fatty liver	[192]
Butyrate (400 mg/kg/day)	Yes/Yes	Maternal diabetes	NOD mice/M	16	Diabetes	[193]
Butyrate (400 mg/kg/day)	Yes/Yes	Tryptophan-free diet	SD rat/M	16	Hypertension	[194]
Quercetin (50 mg/kg/day)	Yes/Yes	High-fat diet	SD rat/M and F	14	Obesity, hyperglycemia, hyperlipemia, and hyperinsulinemia	[195]
Melatonin (1 mg/kg/day at night)	No/Yes	Neonatal corticosterone exposure	SD rat/M and F	16	Diabetes, liver steatosis, and hyperlipidemia	[196]
Melatonin (0.01% in drinking water)	Yes/Yes	Low caloric diet	SD rat/M	12	Hypertension and kidney disease	[197]

^1^ Recombinant murine leptin oral solution, containing five times the average amount ingested from maternal milk. SD, Sprague–Dawley rat; M, male; F, female; CKD, chronic kidney disease; PUFA, polyunsaturated fatty acids; LPS, lipopolysaccharide; NOD, non-obese diabetic.

## Data Availability

Data are contained within the article.
